# Seafarers' mental health status and life satisfaction: Structural equation model

**DOI:** 10.3389/fpubh.2022.969231

**Published:** 2022-11-30

**Authors:** Fereshteh Baygi, Andrew Smith, Nami Mohammadian Khonsari, Fatemeh Mohammadi-Nasrabadi, Zohreh Mahmoodi, Armita Mahdavi-Gorabi, Mostafa Qorbani

**Affiliations:** ^1^Research Unit of General Practice, Department of Public Health, University of Southern Denmark, Odense, Denmark; ^2^Centre for Occupational and Health Psychology, School of Psychology, Cardiff University, Cardiff, United Kingdom; ^3^Student Research Committee, Alborz University of Medical Sciences, Karaj, Iran; ^4^Food and Nutrition Policy and Planning Research Department, National Nutrition and Food Technology Research Institute (NNFTRI), Faculty of Nutrition Sciences and Food Technology, Shahid Beheshti University of Medical Sciences, Tehran, Iran; ^5^Social Determinants of Health Research Center, Alborz University of Medical Sciences, Karaj, Iran; ^6^Non-communicable Diseases Research Center, Alborz University of Medical Sciences, Karaj, Iran; ^7^Chronic Diseases Research Center, Endocrinology and Metabolism Population Sciences Institute, Tehran University of Medical Sciences, Tehran, Iran

**Keywords:** seafarer, wellbeing, mental health, physical health, satisfaction with life

## Abstract

**Background:**

A variety of factors influence seafarers' health. Such factors might affect their satisfaction with life.

**Aims:**

To examine the relationships between seafarers' mental health status and satisfaction with life by using a structural equation method.

**Methods:**

In this survey, 470 seafarers were selected *via* convenience sampling method from two shipping companies. Validated questionnaires including Satisfaction with Life Scale (SWLS), generalized anxiety disorder-7 (GAD-7), Post-traumatic Stress Disorder-8 (PTSD-8), Patient Health Questionnaire-9 (PHQ-9), General Health Questionnaire-12 (GHQ-12), Perceived Health status and Depression-Anxiety-Stress scale-21 (DASS-21) were used to assess different aspects of well-being and life satisfaction. The stratified path analysis method was applied to analyze the data.

**Results:**

439 seafarers (200 officers and 237 non-officers) with a mean age of 34.5 (SD: 8.05) participated in the current study. The GHQ score directly affected satisfaction with life in both officers (β = 0.35) and non-officers (β = 0.40). Also, perceived health status directly and indirectly affected satisfaction with life among officers (β = 0.19) and non-officers (β = 0.06). While officers working days per month indirectly impacted satisfaction with life through the general anxiety disorder, perceived health status, depression, anxiety, stress and current mental health. In non-officers, generalized anxiety disorder had the most potent indirect effects on satisfaction with life through perceived health status and current mental health.

**Conclusion:**

Perceived health status, directly and indirectly, affected seafarers' satisfaction with life. Measures should be taken in order to improve seafarers' perceived health status and its effects on satisfaction with life.

## What is already known about this subject

- There is a broad literature on the adverse effects of life dissatisfaction on the overall health of workers in land-based occupations; less is known in maritime settings.
- The focus of a life satisfaction study among seafarers has been on work characteristics (e.g., contract and Internet access) but not on health and well-being.

## What this study adds

- This study provides a deep understanding of life satisfaction and associated factors for shipping companies and other stockholders in the maritime setting to tackle the stressors at sea and provide a healthy workplace.

## What impact this may have on practice or policy

- The knowledge provided by this study might be used to improve seafarers' working conditions, overall health and well-being, which will directly affect their productivity and indirectly affect the economy, environment, and public safety.

## Introduction

Maritime working environment includes many risks, challenges and stressors (1). Seafarers of long-voyage vessels work and live in an isolated environment, away from home, for several months (4–6 months) (2). In such a workplace, employees' overall health and well-being are impacted by the workplace and its stressors (3). So, to tackle the stressors at sea and provide a healthy workplace, examining different factors that might influence overall health, including mental-physical health and well-being (e.g., satisfaction with life) of seafarers at sea, is necessary.

According to the literature, employees who are more satisfied with their life have better performance in many respects, including income, health, job success and productivity (4). Since workplace at sea has a hazardous characteristic, any adverse effect related to job and health can affect the individual health and well-being as well as the economy, environment and public safety (2). So, in a maritime setting, the positive outcomes in connection with health, well-being and job would be highly beneficial for individuals, organizations and the community in the long run.

Although job satisfaction and consequently the satisfaction with the life of the employees should be viewed as a significant factor for the shipping companies to become more successful, little is known on this topic of seafarers of long voyage vessels (5). On the other hand, the focus of such studies (5) mainly concerned the work characteristics (e.g., seafarers' contracts and Internet access). However, this study addresses the following research questions to provide a complete picture of life satisfaction in a maritime setting: 1. how might demographic and work-related characteristics influence satisfaction with life among seafarers? 2. How might mental health influence satisfaction with life among seafarers? Findings from this study will contribute to the sustainable development goals (SDGs) (Goal_3_: Good Health and Well-being). Valid documentation of seafarer's life satisfaction and associated factors will help shipping companies and other stockholders of maritime settings to provide right working conditions to improve seafarer's overall health and well-being which will directly affect their productivity.

## Methods

The sample size was determined according to a previous study (6). Considering the prevalence of anxiety among seafarers of 17% and error type I of 0.05 and precision 3.4%, the sample size was estimated to be 470 subjects.

This cross-sectional study was done on 473 multinational seafarers working on international oil tankers of two shipping companies. Within the first half of the year 2020, several invitations were sent to all crew members regardless of their rank and position; due to our follow-up and persistence, we had a participation rate near to 93.5%. Thus, nearly all crew member working in the shipping company was assessed.

Objectives and all other aspects of the study were explained to those who wished to participate. Signed informed consent was obtained from all participants, and the participants were assured that their information would be anonymous and not be shared with any third parties.

Demographic and work-related characteristics such as age, marital status, position and duties on the ship, working days and hours and ship characteristics were gathered by using self-administrated questionnaires. Mental health status and life satisfaction evaluations were also obtained *via* validated online self-administrative questionnaires. In these questionnaires thorough explanations were implemented for the participants. It should be noted that all questionnaires were in English, and all participants could read and understand English as well.

The mental health status of the participants was evaluated by validated questionnaires. Anxiety, depression, and stress was assessed by Depression-Anxiety-Stress Scale (DASS-21) (7), General Health Questionnaire-12 (GHQ-12) (8), Post-traumatic Stress Disorder (PTSD-8) (9) and General Anxiety Disorder (GAD-7) (10).

DASS-21 is comprised of 21 questions, and the participant may choose one of the four options in each question. Then the scores are summed. Scores above 7, 9 and 14 in each domain are considered possible disorders in the aforementioned domain (7).

GHQ-12 is mainly used for the determination of general psychiatric disorders. The response of each question was a Likert scale (1 = less than usual, 2 = no more than usual, 3 = somewhat more than usual, 4 = greatly more than usual), and the total score was the sum of twelve items.

PTSD-8 is a short questionnaire to assess the possible presence of post-traumatic stress disorder symptoms. This questionnaire mainly focuses on intrusion, avoidance, and hyper-vigilance domains of PTSD. The participants had four options to choose from. Items range from 1, meaning “not at all” to 4 meaning “very often.” The overall score was the total score of each question

GAD-7 is a sort of questionnaire in order to detect social anxiety, anxiety, and panic disorders. This seven-item questionnaire gives the participants four options to choose from, ranging from “0 = not at all to 3 = nearly every day”. Based on the sum of all scores, we can surmise the level of anxiety. The scores of 5, 10, and 15 are cut-off points for mild, moderate, and severe anxiety, respectively (9).

Satisfaction with life scale (SWLS) is a popular five-scale questionnaire widely used to evaluate a population's satisfaction with life. The participant can choose one of the seven options, from one meaning “strongly disagree” to seven meaning “strongly agree.” The total score scores range from 5 to 35 (11, 12).

The Perceived health status of the participants was assessed with a single question, asking them to rate their health based on their own opinion, from one as extremely bad to 10 as healthy (13, 14).

Data were analyzed using Lisrel 8.8 and SPSS 22 software. Structure equation model (SEM) was used to assess the association of demographic characteristics, work-related variables, and mental health status with life satisfaction. SEM is a generalized method of multiple regression that, in addition to providing the direct effects, also expresses the indirect effects and the effect of each independent variable on the dependent variables (15). Since in the primary analysis, the association of demographic characteristics, work-related variables and mental health status with life satisfaction according to job title (officer/non-officer) was different, therefore we fitted two different SEMs for officers and non-officers. In the conceptual model of SEM, age, working days per month (DPM) and working hours per week (HPW) were considered as independent variables in the model. It was assumed that these variables affected dependent variables (mental health variables) and SWLS. The normality of continuous variables was assessed using Kolmogrov-smirnov test. The correlation between continuous variables was assessed using Pearson correlation test. The results of SEM were reported as beta (β) coefficient and *T*-value. The level of significance was set at *T*-values > 1.96.

## Results

Of the 437 of our participants, 200 of them were officers with a mean (SD) age of 36.8 (7.7), and 237 were non-officers with a mean age (SD) of 32.5 (7.8). It should be noted that all crew members were male. The characteristics of the participants, alongside the scores of their questionnaires, are presented as Mean ± SD in Table 1.

**Table 1:** Characteristics of the seafarers presented as Mean ± SD.

**Variable**	**Total (*N* = 437)**	**Officer (*N* = 200)**	**Non officer (*N* = 237)**
Age (year)	34.49 ± 8.05	36.8 ± 7.7	32.56 ± 7.82
GAD	1.53 ± 2.7	2.1 ± 3.33	0.97 ± 1.94
HPW	67.29 ± 9.56	67.27 ± 9.62	67.37 ± 9.48
DPM	11.87 ± 4.21	11.27 ± 4.11	12.38 ± 4.24
DASS	4.2 ± 7.22	5.77 ± 8.25	2.78 ± 5.62
PHS	1.44 ± 0.608	1.51 ± 0.62	1.379 ± 0.588
PTSD	11.60 ± 4.74	12.76 ± 5.36	10.54 ± 3.86
GHQ	6.56 ± 5.06	7.95 ± 5.96	5.35 ± 3.66
SWLS	11.27 ± 4.65	12.08 ± 5.17	10.62 ± 4.056

GAD, General anxiety disorder; PHS, Perceived health statues; DASS, Depression anxiety stress scales; PTSD, Post-traumatic stress disorder; GHQ, General health questionnaire; SWLS, Satisfaction with life scale; DPM, Working days per month; HPW, Working hours per week.

Table 2 shows the path standardized and unstandardized coefficients of mental health-related variables and work-related characteristics in officer and non-officer seafarers.

**Table 2:** Standardized and unstandardized path coefficients for variables according to job title.

**Variables**	**Officer**			**Non officer**		
	**β estimate**	**Standardized β estimate**	***T*-value**	**β estimate**	**Standardized β estimate**	***T*-value**
DPM→ PHS	0.02	0.14	2^*^	–	–	–
DPM→ GAD	0.17	0.21	4.28^*^	–	–	–
DPM→ DASS	0.29	0.14	2.05^*^	–	–	–
HPW→ PHS	–	–	–	0.01	0.13	2.19^*^
HPW→ DASS	–	–	–	0.08	0.13	2.07^*^
HPW→ PTSD	–	–	–	−0.04	−0.10	1.72
HPW→ GHQ	–	–	–	−0.02	−0.05	0.89
PHS→ GHQ	1.25	0.13	2.89^*^	0.96	0.15	2.68^*^
PHS→ SWLS	1.25	0.15	2.18^*^	0.05	0.01	0.11
GHQ→ SWLS	0.30	0.35	4.97^*^	0.44	0.40	6.48^*^
GAD→ GHQ	1.11	0.62	9.98^*^	0.6	0.31	4.81^*^
GAD→ PHS	0.51	0.27	3.89^*^	0.09	0.30	4.81^*^
DASS→ GAD	0.23	0.56	9.43^*^	0.08	0.23	3.74^*^
DASS→ PTSD	0.37	0.57	2.05^*^	0.33	0.47	8.17^*^
DASS→ GHQ	0.083	0.11	1.94	–	–	–
PTSD→ GAD	0.11	0.17	2.9^*^	0.19	0.37	5.95^*^
PTSD→ GHQ	–	–	–	0.25	0.26	4.27^*^

* Significant at *p* < 0.05.

GAD, General anxiety disorder; PHS, Perceived health status; DASS, Depression anxiety stress scales; PTSD, Post-traumatic stress disorder; GHQ, General health questionnaire; SWLS, Satisfaction with life scale; DPM, Working days per month; HPW, Working hours per week.

**Figure 1:**
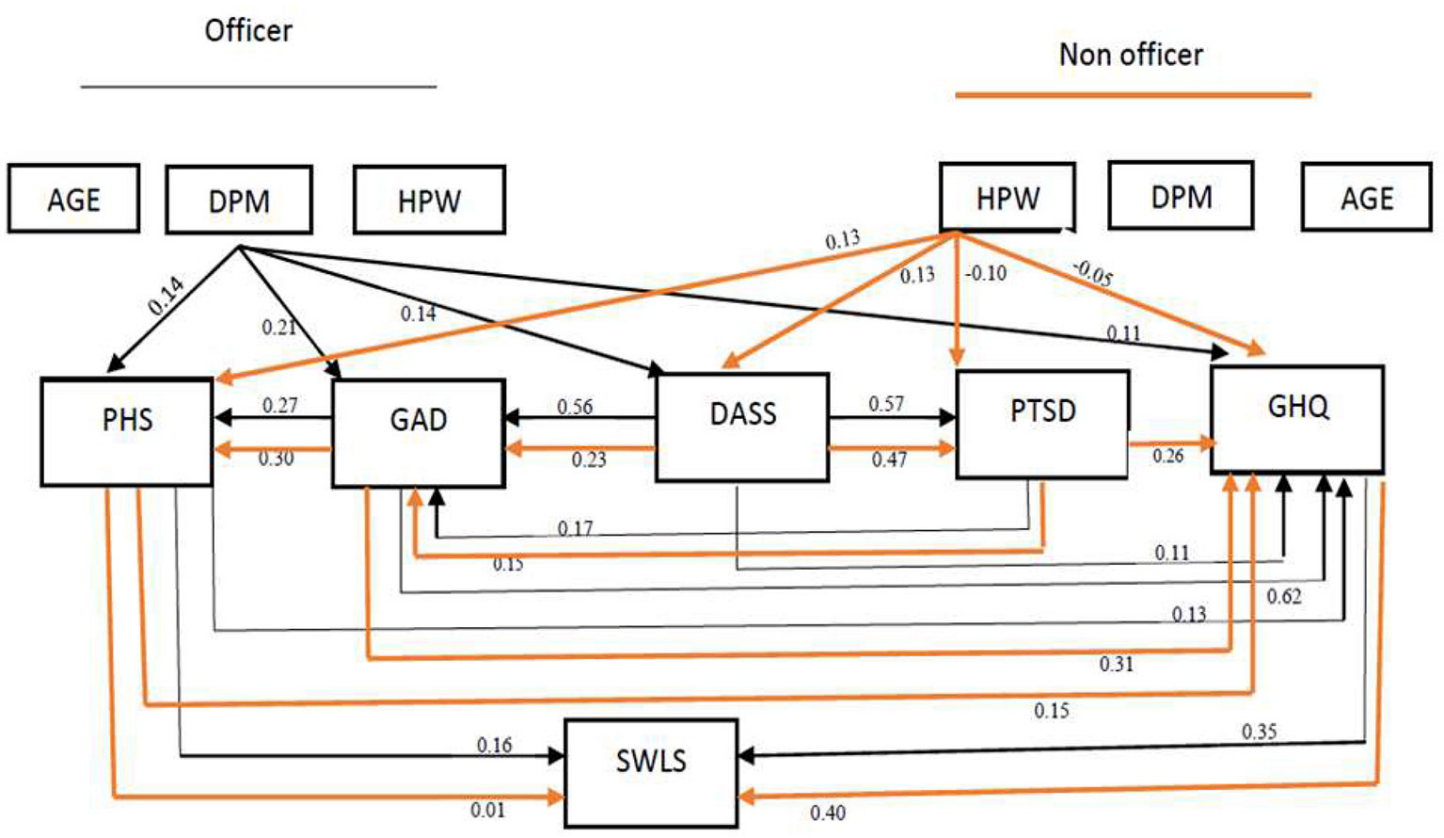
Path diagram for the association of age, work related characteristics and mental health status with life satisfaction in seafarers. GAD, General anxiety disorder; PHS, Perceived Health Status; DASS, Depression Anxiety Stress Scales; PTSD, Post-traumatic Stress Disorder; GHQ, General Health Questionnaire; SWLS, Satisfaction with Life Scale; DPM, Working days per month; HPW, Working hours per week.

Figure 1 shows the path diagram for the association of age, work-related characteristics and mental health status with life satisfaction in seafarers. In the officer group, GHQ was the only variable with a significant and positive relationship with SWLS *via* a singular direct path (β = 0.35). In the indirect path, DPM had the most positive and significant correlation with SWLS vis effect on PHS, GAD, DASS, and GHQ. Moreover, PHS was the only variable with a significant relationship with SWLS *via* direct and indirect pathways (β = 0.19). In the non-officer group, GHQ was the only variable with a significant and positive correlation with SWLS *via* a singular direct path (β = 0.40). Moreover, GAD indirectly had the most significant and positive correlation with SWLS *via* effect on GHQ and PHS (β = 0.17). In officers, PHS is the only variable which is correlated with SWLS *via* both direct and indirect pathway (β = 0.06) (Table 2).

Table 3 shows the direct, indirect, and total effect of work-related and mental health variables on SWLS according to job title (officer and non-officer). In officers, the strongest significant effect was observed between DPM and SWLS (β = 0.37) and in non-officers, the highest total effect was observed between GHQ and SWLS (β = 0.40).

**Table 3:** Total effect of variables on satisfaction with life scale according to job title.

**Variables**	**Officer**			**Non-officer**		
	**Direct effect**	**Indirect effect**	**Total effect**	**Direct effect**	**Indirect effect**	**Total effect**
DPM	–	0.378^*^	0.378^*^	–	–	–
HPW	–	–	–	0.11	0.013^*^	0.013^*^
PHS	0.15^*^	0.045^*^	0.19^*^	0.01	0.06^*^	0.06^*^
GAD	–	0.269^*^	0.269^*^	–	0.17^*^	0.17^*^
DASS	–	0.121^*^	0.121^*^	–	0.11^*^	0.11^*^
PTSD		0.021^*^	0.021^*^	–	0.16^*^	0.16^*^
GHQ	0.35^*^	–	0.35^*^	0.40^*^	–	0.40^*^

* Significant at *p* < 0.05.

GAD, General anxiety disorder; PHS, Perceived health status; DASS, Depression anxiety stress scales; PTSD, Post-traumatic stress disorder; GHQ, General health questionnaire; SWLS, Satisfaction with life scale; DPM, Working days per month; HPW, Working hours per week.

The results of the model fit indices according to job title (officer and non-officer) are presented in Table 4. Based on the results in both groups, the model had acceptable fitness.

**Table 4:** The fitness of model according to job title.

	**X^2^**	**df**	**CFI**	**GFI**	**NFI**	**RMSEA**	**IFI**
Officer	6.30	8	1	0.99	0.99	< 0.001	1
Non-officer	12.92	7	0.99	0.98	0.97	0.06	0.99

Df, degree of freedom; CFI, Comparative fit index; GFI, Goodness of fit; NFI, Normed-fit index; RMSEA, Root mean square error of approximation; IFI, Incremental fit index.

## Discussion

The most direct factors affecting SWLS were PHS and GHQ in both officers and non-officers. However, PHS in officers was affected by DPM, whereas GHQ in non-officers was affected by HPW. Other theoretical determinants of SWLS, including GAD, DASS, and PTSD, are affected by DPM and HPW in officers and non-officers, respectively. In other words, the most important factor influencing life satisfaction, perceived health, depression, anxiety, and stress is work hours in officers and workdays in non-officers. In a study on Croatian seafarers, who were officers and employed on cargo ships, job and life satisfaction levels were higher for the shorter duration onboard and a favorable ratio of work to non-workdays (5). The onboard psychophysical stress is essentially determined by the working hours/day in Oldenburg and Jensen study, to (13). Shift, long working days, irregular working hours, and lack of sleep were classified as the main psychosocial stressors in seafarers based on confirmatory factor analysis by Rengamani and Murugan (14).

The non-officers got less scores in GAD, DASS, and PTSD as the mental health assessment questionnaires. However, except for DPM and HPW, significant differences were not observed in the present study's variables affecting life scale between officers and non-officers. Oldenburg and Jensen (16) recently found a high prevalence of over-commitment, particularly among officers, that can lead to mental exhaustion.

An average working time of nearly nine and a half hours daily on-board is equal to a working week of nearly 66 h, as a ship is in continuous operation, primarily if it operates in a coastal area. This high amount of working hours per week means that even at weekends and on public holidays, work must be done mainly if the ship is in port and the mooring fees for the shipping company are very high (13). Working tasks and stress levels of seafarers of container ships depend on the voyage episode (15). It is well known that exceptionally long working hours, interacting with various occupational factors, can impact workers' health, physiologically and psychologically (17, 18).

In a French seafarers survey on oceanographic vessels, more than one-third of seafarers reported mental stress in the overall health tests (19). Most seafarers were spending months or more on-board away from home with loneliness, bullying, and fatigue (20). A unique risk factor among officers is the accumulation of administrative work in the port, which also disrupts the practical organization of work (21). Therefore, seafaring is included in the high-risk occupational groups for stress (22) and other mental health outcomes (23), such as anxiety and depression (20). In other words, psychological disorders like depression, anxiety, suicide, and alcohol or drug dependency, are well-recognized health related problems within the maritime settings (24).

Seafaring is heterogeneous in terms of socio-demographic and working characteristics e.g., age, nationality, duration of stay on board, rank and position on-board, demands, control, support, and the physical environment. Such factors may influence how stress is differentially experienced and affected on quality of life of seafarers. studies suggest that some variables beyond job factors, including dispositional resilience and instrumental work support may also be impacting on psychosocial well-being of merchant seafarers (25, 26). However, social support was significantly associated with health-related quality of life in Chinese seafarers. They were not satisfied with their quality of life more than the general population, especially in terms of availability of money for their needs, leisure-time physical activity, medical treatment, and availability of information to meet daily needs (27). Besides, Chinese seafarers reported lower quality of life in the four domains (the physical health, psychological health, social relations, and environment domains) compared to Polish seafarers (28).

Existing studies about mental health status of the international seafarers have been limited due to difficulties in obtaining information and comparison with other workers or overtime. Mental health and the health of seafarers are of considerable concern to maritime authorities, employers' associations, and trade unions of this filed. However, employers do not see this as an immediate problem. Studies found that some shipping companies apply preventive measures in support of the mental health and well-being on board. Examples of these actions include: improving the ship's communications means and existing entertainment facilities, seafarers' employment conditions and physical health. These measures are likely to be more effective in improving the seafarers' mental health and well-being than current response strategies (e.g., advising seafarers) and self-help strategies for seafarers (29).

Preventive measures should be taken to reduce stress and other health effects in a maritime setting (3). In addition, the need for improvement and prevention may vary between different groups of seafarers, with higher leadership responsibilities among officers and greater physical demand among lower crews (17). Prevention, introductory training and training courses should be aimed at maximizing the awareness of seafarers and encouraging the use of specialized programs developed for welfare. Knowing the first mental changes of seafarers, ways of self-help and social development is the next step. The emotional intelligence of investing in the well-being of the people certainly creates a competitive advantage in the transport company; Moreover, it directly impacted the turnover and career prospects of young people who have chosen one of the most challenging and dangerous but significant jobs (30). The main intervention strategies in reducing stress and occupational health risks among seafarers should focus on reducing the central occupational pressures and risks (initial measures). They can also be considered as third-degree (coping with stress outcomes) or in some cases, secondary intervention measures (helping to deal with stressors) (31).

GHQ had the most significant direct impact on SWLS in both officers and non-officers, DPM had the most significant indirect impact on SWLS in officers, whereas GAD had the most significant indirect path on SWLS. Thus, a variety of simple questionnaires can be used to evaluate the workers' sense of well-being (Life satisfaction) to provide better support and care.

Mental health support strategies should be directed toward the success of the ship's activities, which are designed for positive social stimulation and in case of relaxation, recharging and raising the morale of the sailors. Terms and conditions are also necessary to support a right balance between sailors' lives and work (29).

## Limitations and strengths of the study

The cross-sectional nature of the study is the main limitation. There may be some unobservable and unmeasured factors not included in our model, which might influence on life satisfaction, too. There is a bi-directional link between the life satisfaction and mental health status which makes it difficult to assess by the one-way relationship used in structural equation modeling (24, 30). So, further studies to examine the effects of life dissatisfaction on mental health are recommended. Also, the authors believe that the role of seafarers' contracts and Internet access- as important work characteristics- on their life satisfaction should be assessed at both individual and organizational level. But in the current study, the shipping companies disagreed to assess organizational perspective. So, we suggest that researchers consider this on their future studies.

To our knowledge, the current study is the first project to investigate the patterns of effects on seafarers' sense of well-being alongside identifying predictors of stress and life satisfaction in a sample of seafarers- working on long voyage tankers- using a structural equation model.

## Conclusion

Perceived health status, directly and indirectly, affected seafarers' satisfaction with life. Other important factors influencing life satisfaction, perceived health, depression, anxiety, and stress is work hours in officers and workdays in non-officers. Measures should be taken in order to improve the seafarers' health conditions and perceived health status and its effects on satisfaction with life. Furthermore, longitudinal studies on the work-related demands of shipboard officers and crews are recommended to determine possible chronic health impacts.

## Data availability statement

The raw data supporting the conclusions of this article will be made available by the authors, without undue reservation.

## Ethics statement

The studies involving human participants were reviewed and approved by Alborz University of Medical Sciences. The patients/participants provided their written informed consent to participate in this study.

## Author contributions

MQ conceived the study, participated in study design, data collection, and data analysis. FB conceived the study, participated in study design and data collection, interpretation of the result and wrote the manuscript. ZM and NM participated in data collection and data analysis. FM-N and AM-G assisted with the preparation of the manuscript and interpretation of the results. AS conceived the study and revised the manuscript critically. All the authors have read and approved the final submitted manuscript.

## Funding

Alborz University of Medical Sciences funded this study, Iran (Grant No. 99.51) as a joint project with the scientific contribution of the University of Southern Denmark.

## Conflict of interest

The authors declare that the research was conducted in the absence of any commercial or financial relationships that could be construed as a potential conflict of interest.

## Publisher's note

All claims expressed in this article are solely those of the authors and do not necessarily represent those of their affiliated organizations, or those of the publisher, the editors and the reviewers. Any product that may be evaluated in this article, or claim that may be made by its manufacturer, is not guaranteed or endorsed by the publisher.
